# 

**Published:** 1895-03-09

**Authors:** 


WITH EXTRA NURSING SUPPLEMENT.

Per Annum, 10s. 6d., post free.

Monthly Parts, 6d.; post free, 8d.

Ditto per Annum, 8s. 6d., post free.

### THE HOSPITAL

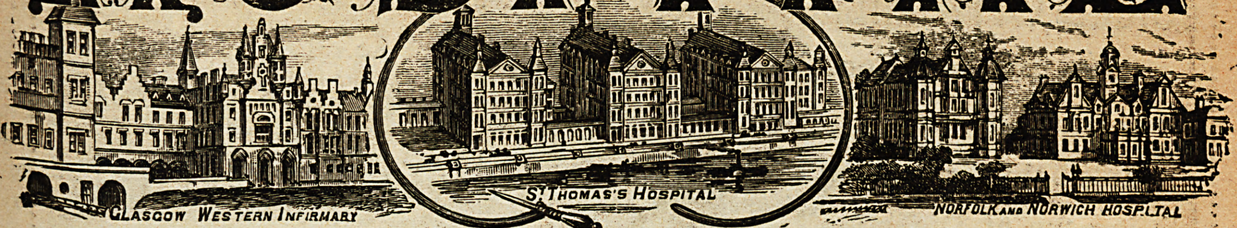

No. 441. Vol. XVII. WITH EXTRA NURSING SUPPLEMENT. Saturday, March 9th, 1895.

